# Is PCR the Next Reference Standard for the Diagnosis of *Schistosoma* in Stool? A Comparison with Microscopy in Senegal and Kenya

**DOI:** 10.1371/journal.pntd.0003959

**Published:** 2015-07-28

**Authors:** Lynn Meurs, Eric Brienen, Moustapha Mbow, Elizabeth A. Ochola, Souleymane Mboup, Diana M. S. Karanja, W. Evan Secor, Katja Polman, Lisette van Lieshout

**Affiliations:** 1 Department of Parasitology, Leiden University Medical Center, Leiden, The Netherlands; 2 Department of Biomedical Sciences, Institute of Tropical Medicine, Antwerp, Belgium; 3 Laboratory of Bacteriology and Virology, Aristide Le Dantec Teaching Hospital, Dakar, Senegal; 4 Center for Global Health Research, Kenya Medical Research Institute, Kisumu, Kenya; 5 Division of Parasitic Diseases and Malaria, Centers for Disease Control and Prevention, Atlanta, Georgia, United States of America; National Institute of Parasitic Diseases, CHINA

## Abstract

**Background:**

The current reference test for the detection of *S*. *mansoni* in endemic areas is stool microscopy based on one or more Kato-Katz stool smears. However, stool microscopy has several shortcomings that greatly affect the efficacy of current schistosomiasis control programs. A highly specific multiplex real-time polymerase chain reaction (PCR) targeting the *Schistosoma* internal transcriber-spacer-2 sequence (ITS2) was developed by our group a few years ago, but so far this PCR has been applied mostly on urine samples. Here, we performed more in-depth evaluation of the ITS2 PCR as an alternative method to standard microscopy for the detection and quantification of *Schistosoma* spp. in stool samples.

**Methodology/Principal findings:**

Microscopy and PCR were performed in a Senegalese community (n = 197) in an area with high *S*. *mansoni* transmission and co-occurrence of *S*. *haematobium*, and in Kenyan schoolchildren (n = 760) from an area with comparatively low *S*. *mansoni* transmission. Despite the differences in *Schistosoma* endemicity the PCR performed very similarly in both areas; 13–15% more infections were detected by PCR when comparing to microscopy of a single stool sample. Even when 2–3 stool samples were used for microscopy, PCR on one stool sample detected more infections, especially in people with light-intensity infections and in children from low-risk schools. The low prevalence of soil-transmitted helminthiasis in both populations was confirmed by an additional multiplex PCR.

**Conclusions/Significance:**

The ITS2-based PCR was more sensitive than standard microscopy in detecting *Schistosoma* spp. This would be particularly useful for *S*. *mansoni* detection in low transmission areas, and post-control settings, and as such improve schistosomiasis control programs, epidemiological research, and quality control of microscopy. Moreover, it can be complemented with other (multiplex real-time) PCRs to detect a wider range of helminths and thus enhance effectiveness of current integrated control and elimination strategies for neglected tropical diseases.

## Author Summary

In the developing world, over 207 million people are infected with parasitic *Schistosoma* worms. *Schistosoma mansoni* is one of the most widespread species, and its routine diagnosis is based on microscopic detection of parasite eggs in stool samples. This technique is, however, highly observer-dependent and has suboptimal sensitivity. We compared the performance of stool microscopy with the highly specific real-time polymerase chain reaction (PCR) we recently described for the detection and quantification of parasite–specific DNA. We tested stool samples collected at two different epidemiological settings: a Senegalese population (n = 197) from a high transmission area where *S*. *mansoni* and *S*. *haematobium* are co-endemic and a Kenyan school population (n = 760) selected from zones with comparatively low *S*. *mansoni* transmission. Microscopy mostly missed low intensity infections that PCR was able to detect. Consequently, the PCR may be very useful for the detection of *S*. *mansoni* in areas with low levels of infection. Furthermore, being a highly standardized diagnostic procedure, the PCR may improve schistosomiasis control programs, epidemiological research, and quality control of microscopy. Also it can be easily combined with other PCRs to detect a wider range of helminth infections in a single stool sample.

## Introduction

Schistosomiasis control strategies are currently based on mass drug administration (MDA) with praziquantel to populations at risk [1]. Disease mapping, MDA allocation, and post-MDA monitoring of infection are based on standard microscopy techniques: urine filtration for *Schistosoma haematobium*, and Kato-Katz on stool for the other *Schistosoma* spp., including *S*. *mansoni*. However, these techniques are laborious and there are recognized deficiencies in their sensitivity, thereby limiting the accuracy of screening and monitoring results, and thus appropriate decision-making [2]. This impairs the efficiency of global efforts to control and eventually eliminate schistosomiasis.

Better diagnostics have great potential to improve the quality of schistosomiasis control programs. For *S*. *haematobium*, a good alternative to standard microscopy is already available in the form of hematuria dipstick tests [3]. The diagnosis of *S*. *mansoni* however, still heavily relies on the Kato-Katz thick stool smear. Several other detection tools have been proposed, including the circumoval precipitin test on serum samples [4,5], the FLOTAC technique on fecal samples [6], and the point-of-care circulating cathodic antigen assay (POC-CCA) for detection of *Schistosoma* antigen in urine samples [7,8]. In addition, DNA-based methods, such as real-time polymerase chain reaction (PCR)-based techniques, are increasingly being used for the detection of *Schistosoma* spp. infections [9–18]. The advantage of microscopy over *Schistosoma* species-specific antigen tests is that they can detect multiple helminth species, and that they are quantitative. These features make them better apt for large-scale use in integrated neglected tropical disease (NTD) control programs than the single-pathogen tests. PCR, in a multiplex format, has the same above-mentioned advantages as microscopy but has greater flexibility. Indeed, a multiplex PCR can detect all (*Schistosoma* and other helminth) species at the same time, and at any moment after the stool has been collected. Moreover, PCR is a highly standardized diagnostic procedure and it can also be used to detect parasitic protozoa or other microorganisms that cannot be identified by Kato-Katz.

The aim of the present study was to compare Kato-Katz with PCR for the detection of *Schistosoma*—and soil-transmitted helminth (STH)—infections in stools from persons living in *S*. *mansoni*-endemic areas. To this end, stool samples from ongoing studies in two countries with different endemicity were examined using both tests.

## Materials and Methods

### Ethics statement

Informed and written consent was obtained from all participants prior to inclusion into the study. For minors, informed and written consent was obtained from their legal guardians and assent was obtained from the children. The Senegalese survey was part of a larger investigation on the epidemiology of schistosomiasis and innate immune responses (SCHISTOINIR) for which approval was obtained from the review board of the Institute of Tropical Medicine, the ethical committee of the Antwerp University Hospital and ‘Le Comité National d’Ethique de la Recherche en Santé’ of Senegal. All community members were offered praziquantel (40 mg/kg) and mebendazole (500 mg) treatment after the study according to WHO guidelines [19]. The Kenyan survey was performed within the framework of the Schistosomiasis Consortium for Operational Research and Evaluation (SCORE). Ethical clearance from this study was obtained from the Scientific Steering Committee of the Kenya Medical Research Institute (KEMRI-SSC no. 1768), the Kenyan Ethical Review Committee, and the Institutional Review Board of the Centers for Disease Control and Prevention in the USA. All children who were positive for *Schistosoma* infection were treated with praziquantel (40 mg/kg), and those positive for STHs were treated with albendazole (400mg).

### Study areas

Samples were derived from one community-wide study population from a *S*. *mansoni* and *S*. *haematobium* co-endemic area in northern Senegal with high *S*. *mansoni* transmission [20–22], and from a population of schoolchildren living in a *S*. *mansoni* mono-endemic area with comparatively low transmission in western Kenya [23]. The Senegalese survey was conducted in Ndieumeul and Diokhor Tack, two neighboring communities on the Nouk Pomo peninsula in Lac de Guiers (Guiers Lake). Details on this study area have been described elsewhere [20–22]. Stool and urine samples were collected between July and October 2009 and stool samples for PCR were stored for each participant. Stool samples from a subsample of 197 individuals with complete parasitological data were analyzed by PCR. The Kenyan survey was conducted in the Asembo division of the Rarieda district along the shores of Lake Victoria in western Kenya, within the framework of a larger study on the distribution of *S*. *mansoni* amongst school children. Eight to twelve-year-old children attending public primary schools within a 10km from the lake were included (12km wide transect). In this area, *S*. *haematobium* is virtually absent. Stool samples were collected between October 2010 and April 2011, preferentially from the lower prevalence zones [23], and PCR was performed in a subsample of 760 children from 40 schools with complete parasitological data (see also S1 STARD Checklist).

### Diagnosis by microscopy

In Senegal, two stool and two urine samples were collected from each participant on consecutive days. From each stool sample, a duplicate 25 mg Kato-Katz slide was prepared for quantitative detection of *Schistosoma* spp. eggs and qualitative diagnosis of STHs *Ascaris lumbricoides* and *Trichuris trichiura* by microscopy [24–26]. Duplicate slides were examined by two different technicians >24h after preparation of the Kato-Katz smear, and for *S*. *mansoni* the average egg count was calculated. In addition, filtration of 10 ml of urine was performed using a 12 μm pore-size filter (Isopore, USA) according to standard procedures to detect *S*. *haematobium* eggs [25]. Urine filters were read by a single technician. In Kenya, three stool samples were collected on consecutive days, and from each sample, duplicate 42 mg Kato-Katz slides were prepared for microscopy. *Schistosoma mansoni* was diagnosed quantitatively at least 24h after slide preparation. STHs were diagnosed qualitatively: *A*. *lumbricoides* and *T*. *trichiura* at 24h after slide preparation, and hookworm within 1h of slide preparation. Each slide was examined by two independent microscopists and the average was recorded. Urine filtration was not performed in Kenya. In both countries, microscopy was performed blinded to previous results, and *S*. *mansoni* infection intensity was expressed as the number of eggs detected per gram of feces (epg). Egg-based microscopy results were compared to DNA-based PCR results.

### Diagnosis by PCR

Real-time PCR was performed blinded to previous results. During preparation of the first stool sample, an additional amount of fecal material (~0.7ml) was sieved and diluted in 2ml of 96% ethanol [12]. Samples were frozen, transported to the Netherlands, and stored for weeks to months until PCR analysis was performed at the Leiden University Medical Center. Washing of samples, DNA isolation and the setup of the PCR were performed with a custom-made automated liquid handling station (Hamilton, Bonaduz, Switzerland).

For DNA isolation, 200μl of feces suspension was centrifuged and the pellet was washed twice with 1ml of phosphate-buffered saline. After centrifugation, the pellet was resuspended in 200μl of 2% polyvinylpolypyrolidone (Sigma) suspension and heated for 10 min at 100°C. After sodiumdodecyl sulfate–proteinase K treatment (2h at 55°C), DNA was isolated using QIAamp DNA-easy 96-well plates (QIAgen, Limburg, the Netherlands). In each sample, 10^3^ PFU/mL Phocin Herpes Virus 1 (PhHV-1) was included within the isolation lysis buffer [27,28].

A *Schistosoma* multiplex real-time PCR (Schisto-PCR) was performed as described previously [29], with some minor modifications [30]. This PCR targets the *Schistosoma*-specific internal transcriber-spacer-2 (ITS2) sequence of *S*. *mansoni*, *S*. *haematobium*, and *S*. *intercalatum*, as well as PhHV-1 as an internal positive amplification control. The ITS2-based PCR has been validated extensively with a panel of well-defined DNA and stool sample controls and is virtually 100% specific [30]. Amplification was performed by heating samples for 15 minutes at 95°C, followed by 50 cycles, each of 15 seconds at 95°C and 60 seconds at 60°C. Another multiplex real-time PCR, the ANAS-PCR [31], was performed for the detection of STHs *Ascaris lumbricoides*, *Necator americanus*, *Ancylostoma duodenale* and *Strongyloides stercoralis*. In contrast to the ANAS-PCR, the Schisto-PCR was not designed to differentiate between the different species tested.

Amplification, detection and data analysis were performed with the CFX96 Real-Time System version 1.1 (Bio-Rad, Hercules, CA) [29]. Negative and positive control samples were included in each PCR run. The PCR output from this system consisted of a cycle-threshold (C_t_) value, representing the amplification cycle in which the level of fluorescent signal exceeded the background fluorescence. Hence, low C_t_ values correspond to high parasite-specific DNA loads in the sample tested, and vice versa. The maximum C_t_ value was 50, and indicated DNA-negative stool samples. The C_t_ values of the internal PhHV-1 control were within the expected range for all samples, indicating that there was no evidence of inhibition of amplification in any of these samples.

### Data analysis

IBM SPSS 22.0 (SPSS, Inc.) was used for statistical analyses (see also S1 Dataset and S1 SPSS Syntax). Results were considered significant when the *p*-value was <0.05. Kappa (κ) values were calculated as follows to obtain the level of agreement between microscopy and PCR results beyond that which may be obtained by chance:

$$\kappa =\frac{\text{observed test agreement}\;-\;\text{expected test agreement}}{1\;-\;\text{expected test agreement}}$$

Standard cut-off values were used for egg-based infection categories [1]: *Schistosoma mansoni* infections with 1–99 epg were classified as light-intensity, those with 100–399 epg as moderate, and those with ≥400 epg as heavy-intensity infections. DNA loads as reflected by C_t_-values were not normally distributed. Consequently, the Mann-Whitney U test was used to determine differences in DNA loads between *S*. *mansoni* egg-negative and *S*. *mansoni* egg-positive individuals, and the Kruskal-Wallis test to determine differences in DNA loads between the different egg-based infection categories. Spearman’s rank correlation coefficients were calculated to investigate the correlation between egg- and DNA-based infection intensities, which did not show a linear trend.

In the Senegalese study subjects, we investigated whether PCR outcomes were influenced by *S*. *haematobium* infection status. The Pearson Chi² test (with continuity correction) was used to compare PCR positivity between those with and without *S*. *haematobium* infection. The Mann-Whitney U test was used to compare DNA loads in stool samples between individuals with and without *S*. *haematobium* eggs in urine, as well as between individuals with single *S*. *mansoni* and with mixed *Schistosoma* infections stratified according to *S*. *mansoni* infection intensity.

For the analysis of the Kenyan data at the school level, only schools with data on ≥15 children were included (i.e. 24/40 schools). Pearson’s correlation coefficients were calculated to investigate the correlation between egg- and DNA-based infection prevalences in the different schools. Schools were classified into three groups according to their distance from the shore of Lake Victoria: A) the highest prevalence zone ≤1200m from the lake; B) moderate prevalence zone 1200-3800m from the lake; and C) lowest prevalence zone >3800m away.

## Results

### *Schistosoma mansoni* infection frequencies

When only the first stool sample was taken into account, microscopy detected *S*. *mansoni* infections in 57.4% of subjects in Senegal and in 19.2% of subjects in Kenya (Table 1) whilst PCR detected *Schistosoma* DNA in 72.6% and 32.4% of subjects, respectively. Thus, in Senegal, the Schisto-PCR detected 15.2% ((143–113)/197) more infections than microscopy, and in Kenya, 13.2% ((246–146)/760) more infections than microscopy. When two stool samples were taken into account, 68.5% and 25.9% *S*. *mansoni*-positives were detected by microscopy in Senegal and Kenya, respectively. When three stool samples were taken into account in Kenya, 29.5% *S*. *mansoni*-positives were detected by microscopy. While the percentages of *S*. *mansoni*-positives detected by microscopy increased with an increasing number of stool samples, they were still lower than those detected by Schisto-PCR in a single stool sample, in both countries.

**Table 1:** Percentages of *S*. *mansoni*-positives and infection intensities: Microscopy *versus* PCR.

Diagnostic method	Number of stool sample(s) taken into account ^a^	Percentage of positives (n)		Median infection intensity (range) ^b^	
		Senegal	Kenya	Senegal	Kenya
**Microscopy**	**1**	57.4% (113/197)	19.2% (146/760)	200 (20–3120) epg	18 (6–1482) epg
	**2**	68.5% (135/197)	25.9% (197/760)	200 (10–3470) epg	15 (3–1221) epg
	**3**	N/A	29.5% (224/760)	N/A	12 (2–1164) epg
**PCR**	**1**	72.6% (143/197)	32.4% (246/760)	C_t_ = 22.1 (16.5–37.9)	C_t_ = 24.6 (17.7–38.1)

^a^ Stool samples were collected on consecutive days. In Senegal, a duplicate 25 mg Kato-Katz slide was prepared from each stool sample. Duplicate slides were examined by two different technicians, and the average egg count was calculated. In Kenya, a duplicate 42 mg Kato-Katz slide was prepared from each sample. Each slide was examined by two microscopists and the average was recorded.

^b^ For egg-positive and/or PCR-positive individuals only.

**Fig 1:**
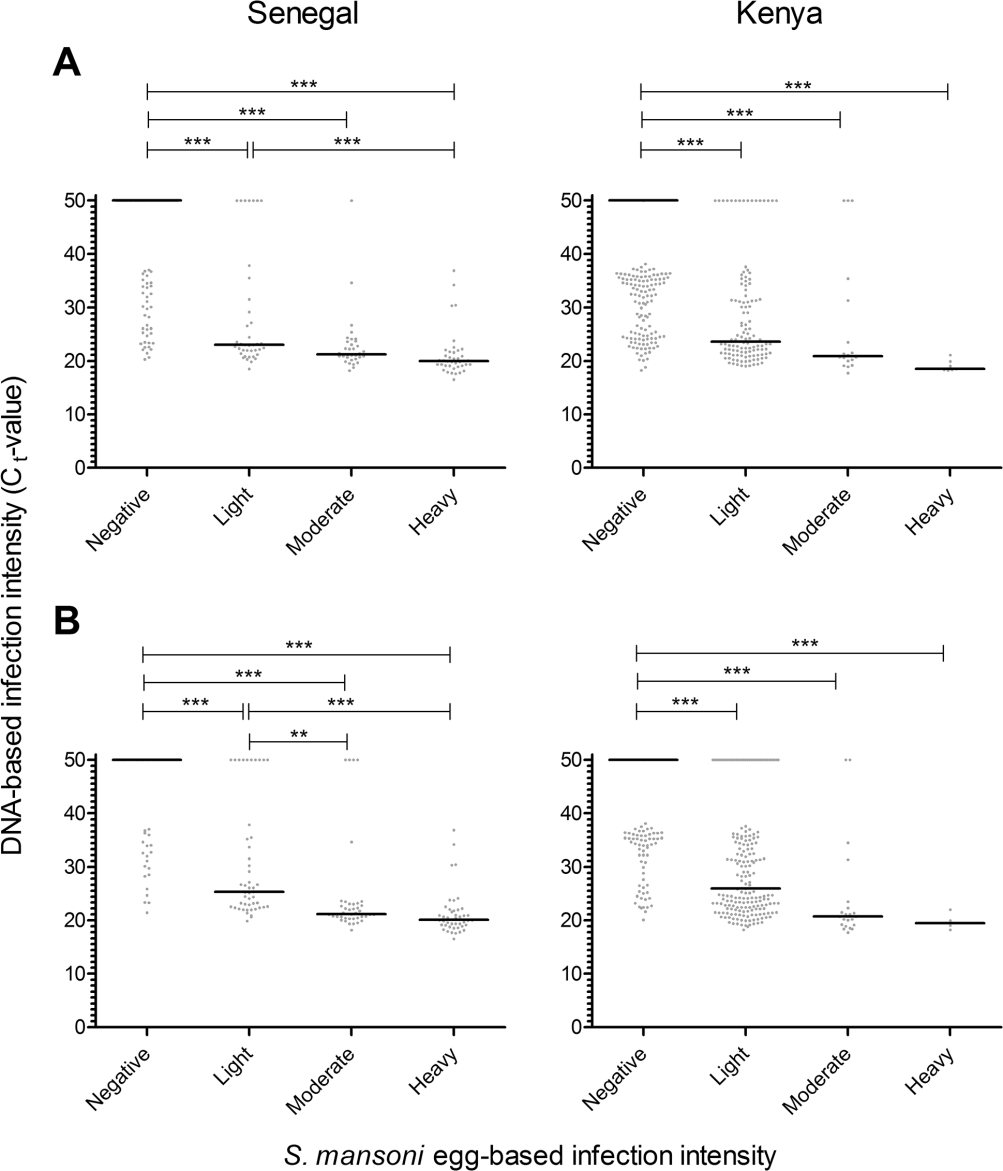
Relationship between egg- and DNA-based *S*. *mansoni* infection intensity. Egg-based infection categories are based on microscopy with standard cut-offs [1]: infections with 1–99 epg were classified as light-intensity, those with 100–399 epg as moderate, and those with ≥400 epg as heavy-intensity infections. DNA-based infection intensity is based on the cycle threshold (C_t_) value of the Schisto-PCR as described in the text. The solid line indicates the median C_t_-value. In **Panel A**, microscopy and PCR were performed on the same stool sample. In Senegal (left panel), 100% (40/40) of people with heavy infection intensities, 97% (33/34) of people with moderate egg counts, and 82% (32/39) of people with light infection intensities according to microscopy were PCR-positive, and in Kenya (right panel) 100% (7/7), 83% (15/18), and 87% (105/121), respectively. In **Panel B**, microscopy was based on either 2 stool samples in Senegal (left panel) or 3 stool samples in Kenya (right panel), while PCR was always based on one stool sample. In Senegal 100% (46/46) of people with heavy infection intensities, 90% (37/41) of people with moderate egg counts, and 79% (38/48) of people with light infection intensities according to microscopy were PCR-positive, and in Kenya 100% (4/4), 91% (20/22), and 80% (159/198), respectively. *p*-values for pairwise comparisons were adjusted for multiple testing. ** p<0.01; *** *p*<0.001.

When based on the first stool sample, egg- and DNA-based results corresponded in 76.6% (κ = 0.500) and 81.8% (κ = 0.536) of subjects in Senegal and Kenya, respectively (Table 2). When egg counts were based on all stool samples provided (2 samples in Senegal and 3 in Kenya), test agreement increased to 81.7% (κ = 0.561), and 86.3% (κ = 0.680), respectively. Differences in test agreement between countries were mainly due to the fact that in Senegal, egg-negatives were more often found positive in PCR than in Kenya (>twofold difference). Fig 1 demonstrates that mainly low-intensity infections were missed when egg counts were based on only one stool sample.

**Table 2:** PCR results in *S*. *mansoni* egg-positives and-negatives.

Country	Microscopy		PCR		Test agreement (κ)
	Number of stool samples taken into account	Egg-based *S*. *mansoni* infection status	Percentage PCR-positives (n)	Median C_t_-value (IQR) ^a^	
**Senegal**	**1**	-	45.2% (38/84)	28.4 (23.4–33.9)	76.6% (0.500)
		+	92.9% (105/113)	21.1 (20.0–23.0)	
	**2**	-	35.5% (22/62)	32.0 (28.2–34.6)	81.7% (0.561)
		+	89.6% (121/135)	21.6 (20.0–23.3)	
**Kenya**	**1**	-	19.4% (119/614)	31.2 (24.2–35.2)	81.8% (0.536)
		+	87.0% (127/146)	22.4 (20.6–26.7)	
	**2**	-	14.4% (81/563)	33.3 (25.2–35.4)	85.1% (0.642)
		+	83.8% (165/197)	23.2 (21.0–29.0)	
	**3**	-	11.8% (63/536)	34.1 (26.3–35.8)	86.3% (0.680)
		+	81.7% (183/224)	23.4 (21.1–30.7)	

^a^ Median and IQR (interquartile range, calculated using Tukey’s hinges) are given for egg-positive and/or PCR-positive individuals only.

### *Schistosoma mansoni* infection intensities

People that were classified as having heavy infections by microscopy were always PCR-positive. Percentages of PCR-positives varied from 97% to 83% in the moderate egg count group, and from 79% to 87% in the group with light intensity infections. Median DNA loads were very similar in both countries for the different *Schistosoma* infection categories (Fig 1).

In both countries, Spearman’s rank correlations between egg- and DNA-based infection intensities were statistically significant (*p*<0.001) with correlation coefficients ranging from -0.638 to -0.782. These correlations became stronger with the number of stool samples that were taken into account (ρ = -0.747 and ρ = -0.782 for 1 and 2 stool samples, respectively, in Senegal; ρ = -0.638, ρ = -0.708, and ρ = -0.738 for 1, 2 and 3 stool samples, respectively, in Kenya).

### Effect of *S*. *haematobium* (co-)infection on PCR results

Based on standard microscopy on stool and urine, 80% (157/197) of the Senegalese subjects were infected with either *Schistosoma* spp. The majority of these infections (92/157) were mixed *S*. *mansoni* and *S*. *haematobium* infections. Single *S*. *mansoni* infections were found in 22%, and single *S*. *haematobium* infections in 11% of subjects (Table 3). Table 3 compares Schisto-PCR outcomes according to *Schistosoma* infection status (by microscopy). DNA-based infection frequencies were highest in those individuals with single *S*. *mansoni* and mixed infections and lowest in persons with single *S*. *haematobium* infections and those without any schistosome infection. As by definition, no *Schistosoma* eggs were observed in stools from uninfected people. In people with single *S*. *haematobium* infections, one would expect a similar (low) percentage of PCR-positives as in uninfected individuals. However, 59% of the single *S*. *haematobium* group was PCR positive, compared to 23% of the microscopy negatives (*p* = 0.009). C_t_-values were comparable. Percentages of PCR-positives were similar in the single *S*. *mansoni* and mixed *Schistosoma* infection groups, but the mixed infection group showed significantly lower C_t_-values (*p* = 0.003), indicative of a higher intensity of infection. No effect of the presence of *S*. *haematobium* on C_t_-values in mixed as compared to single *S*. *mansoni* infections was observed after stratification for egg-based *S*. *mansoni* infection intensity (Table 4).

**Table 3:** PCR results according to *Schistosoma* infection status as assessed by microscopy in Senegal.

Egg-based infection status ^a^	DNA-based results	
	Percentage PCR-positives (n)	Median C_t_-value (IQR) ^b^
**Mixed infections**	89.1% (82/92) ^**d**^	20.9 (19.8–22.9)
**Single *S*. *mansoni***	90.7% (39/43)	22.5 (21.1–25.2)
**Single *S*. *haematobium***	59.1% (13/22) ^**c**^	29.7 (25.9–34.5)
**Negative**	22.5% (9/40)	33.9 (32.6–34.6)

^a^ Based on microscopy on 2 stool samples and 10ml of urine.

^b^ Median and IQR (interquartile range, calculated using Tukey’s hinges) are given for PCR-positive individuals only.

^c^ Including one PCR-negative person with ectopic *S*. *mansoni* eggs in urine, but not in stool.

^d^ Including 14 PCR-positives with ectopic *S*. *mansoni* eggs in both stool and urine, and *S*. *haematobium* in urine.

**Table 4:** The effect of mixed *Schistosoma* infection on C_t_-values in *S*. *mansoni* egg-positive and *Schistosoma* PCR-positive subjects. ^a^

Egg-based *S*. *mansoni* infection intensity	Single *S*. *mansoni*		Mixed *Schistosoma* infection		*p*-value
	n	Median C_t_-value (IQR) ^b^	n	Median C_t_-value (IQR) ^b^	
**Light intensity** (1–99 epg)	20	25.2 (22.8–28.1)	18	22.7 (22.0–26.6)	0.126
**Moderate intensity** (100–399 epg)	10	21.3 (20.7–22.1)	27	21.0 (20.3–22.9)	0.674
**Heavy intensity** (≥400 epg)	9	20.6 (19.4–22.2)	37	20.0 (18.9–21.0)	0.397
**Total**	39	22.5 (21.1–25.2)	82	20.9 (19.8–22.9)	0.003

^a^ Based on microscopy on 2 stool samples and ≥10ml of urine.

^b^ Median and IQR (interquartile range, calculated using Tukey’s hinges) are given for egg-positive and/or PCR-positive individuals only.

### Variation in *S*. *mansoni* infection frequencies between Kenyan schools

**Fig 2:**
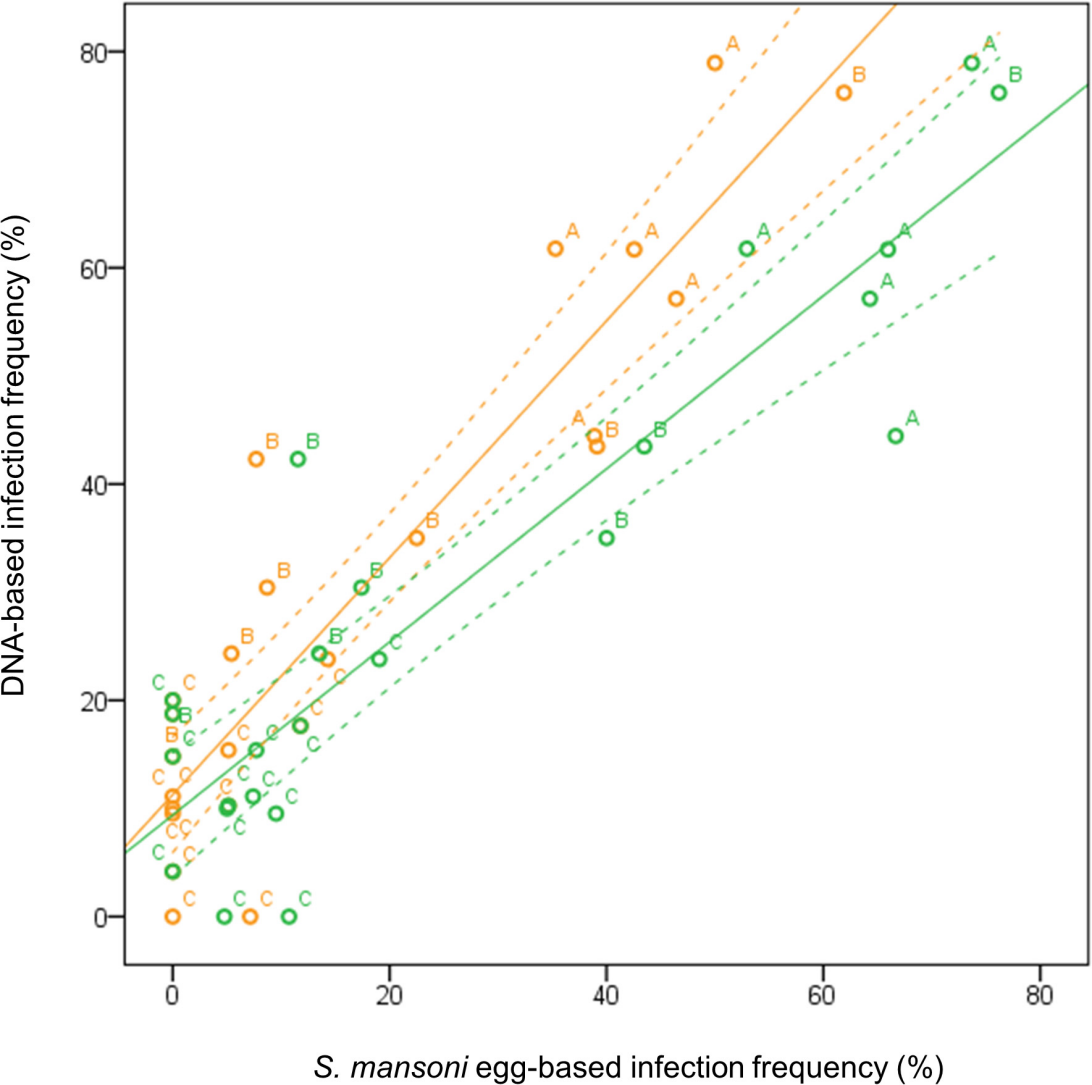
Relationship between egg- and DNA-based *S*. *mansoni* infection frequencies in Kenyan schools. Orange dots refer to infection frequencies based on microscopy performed on one stool sample, and green dots to infection frequencies based on three stool samples. Solid lines indicate regression lines and the dotted lines indicate their corresponding 95% confidence intervals. Schools with samples sizes <15 were excluded. Pearson’s correlation coefficients were 0.850 and 0.826, respectively (both *p*<0.001). ‘A’ indicates schools located within 1200m of the Victoria Lake, ‘B’ those between 1200 and 3800m, and ‘C’ indicates schools located farther than 3800m from the Lake (Foo et al., manuscript in preparation).

To explore the diagnostic value of PCR on stool samples in identifying high-risk schools and/or communities, Kenyan test results were analyzed at school level. Data for 24 schools with at least ≥15 children per school, representing 688 school children, were aggregated. The median sample size per school was 27 (range 15–47). Fig 2 indicates a strong, linear correlation between the percentage of microscopy- and PCR-positives per school (*p*<0.001). DNA-based infection frequencies were consistently higher than egg-based infection frequencies at the school level when both were based on the same stool samples, and PCR identified 25% (22/24 versus 16/24) more *S*. *mansoni-*positive schools than microscopy. When egg counts from all stool samples were taken into account, microscopy identified more *S*. *mansoni*-positive schools (20/24), and also more high-risk schools (infection frequencies ≥50% [1]), as compared to when only the first stool sample was taken into account (Fig 2). In those high-risk schools, egg-based infection frequencies calculated from three stool samples (six slides) were as high as, or higher than DNA-based infection frequencies. In low-risk schools on the other hand (infection frequencies <10%), PCR detected more infections than microscopy on three stool samples, and it detected more positive schools.

### Soil-transmitted helminth infections

In addition to *Schistosoma*, we investigated the occurrence of STH infections by Kato-Katz and ANAS-PCR. In both study areas, microscopy indicated low prevalences of STH infections and this was confirmed by PCR (Table 5). The two techniques detected similar percentages of *A*. *lumbricoides*-positives in both countries. Hookworm was only present in Kenya, and the ANAS-PCR showed that these infections only involved *N*. *americanus*. Interestingly, PCR detected more than threefold the number of hookworm infections than microscopy.

**Table 5:** Diagnosis of soil-transmitted helminths by Kato-Katz and PCR. ^a^

Study site	Helminth species	Percentage egg-positives (n) ^a^	Percentage PCR-positives (n)
**Senegal**	***A*. *lumbricoides***	2.8 (6)	2.4 (5)
	***N*. *americanus***	N/A	0.0 (0)
	***A*. *duodenale***		0.0 (0)
	***S*. *stercoralis***	N/A	0.9 (2)
	***T*. *trichiura***	0.5 (1)	N/A
**Kenya**	***A*. *lumbricoides***	4.3 (33)	6.3 (48)
	***N*. *americanus***	3.3 (25)	12.6 (96)
	***A*. *duodenale***		0.0 (0)
	***S*. *stercoralis***	N/A	0.9 (7)
	***T*. *trichiura***	9.5 (72)	N/A

^a^ Total sample sizes were n = 197 in Senegal and n = 760 in Kenya. Two and three stools samples were taken into account in Senegal and Kenya, respectively.

## Discussion

There are only a handful of studies that compared PCR outcomes with the reference method that is routinely used in endemic areas, i.e. microscopy on Kato-Katz smears [9]. Moreover these studies used different PCR targets [9]. A real-time PCR targeting the cytochrome c oxidase subunit I (*cox*1) of *S*. *mansoni* found similar percentages of *S*. *mansoni*-positives as standard microscopy in a Senegalese population [12]. The sensitivity of this PCR was found to be suboptimal because the *cox*1 region shows considerable genetic variation [32]. PCRs based on the 121-bp tandem-repeat sequence showed more promising results with 7 to 28% higher percentages of *S*. *mansoni* infections detected than standard microscopy [10,13,17,33–37]. In contrast to the ITS2-based real-time PCR used in the present study however [29], this PCR cannot quantify DNA loads. The present study was the first to compare standard microscopy to an improved Schisto-PCR targeting the conserved ITS2 sequence.

The ITS2-based PCR detected 13–15% more *Schistosoma*-positive individuals than microscopy when both tests were performed on the same stool sample. These trends were very similar in the north of Senegal where *S*. *mansoni* prevalences are high [20], and in the west of Kenya using stools from schools that had considerably lower *S*. *mansoni* prevalences [23]. In Kenya, 25% more schools with *S*. *mansoni*-infected children were identified based on PCR as compared to microscopy. We observed that the number of egg-positive individuals increased as more stool samples were taken into account. It is indeed well-known that the sensitivity of microscopy increases as more consecutive stool samples are included in the analysis [38]. This is likely due to the variability of egg counts for an individual with a given worm load [39,40]. More *S*. *mansoni* egg-negatives tested positive in PCR in Senegal than in Kenya. This between-country difference may be due to methodological differences between the two studies, such as the amount of fecal material examined per stool sample. In Senegal, 2x25mg fecal material was examined per stool sample while in Kenya 2x42mg was examined per stool sample and this may have resulted in relatively more false negative microscopy results for *S*. *mansoni* in Senegal. In addition, the co-occurrence of *S*. *haematobium* in the Senegalese population may have resulted in ‘false-positive’ PCR results, as the PCR may pick up some occasional *S*. *haematobium* DNA present in the stools.

Trends for *S*. *mansoni* infection intensities were very similar to those of infection frequencies. While both PCR and microscopy proved adequate to detect *S*. *mansoni* infections with higher egg loads and, consequently higher fecal DNA loads, light infections were more likely to be missed by microscopy. People with light infections often showed low *Schistosoma* DNA levels in stool, and were egg-negative when one stool sample was considered. When more stool samples were tested, these people tended to shift from the negative egg-based infection category towards the light-intensity infection group. Likewise, comparison of the two techniques in Kenya showed that *S*. *mansoni* infections in children from schools with low prevalence and intensity were more likely to be missed by microscopy than those from schools with higher prevalence and intensity. It is indeed known that the sensitivity of microscopy is especially low in light-intensity infections, and in low-transmission areas [40]. Apparently, PCR does not suffer (as much) from this problem and may therefore be particularly useful in such situations. The strong correlation between egg counts and DNA loads in Senegal and Kenya, as well as between egg- and DNA-based infection frequencies in Kenyan schools suggests that DNA loads and DNA-based prevalences can be linked with egg counts and egg-based prevalences, respectively. This implies that the cut-offs which are based on *S*. *mansoni* egg counts and that are currently used for the allocation of control interventions (e.g. for MDA [1]), may be conveniently translated into cut-offs based on fecal *Schistosoma* DNA loads. More studies are needed to assess this into more detail and in more geographical areas [41].

We found the performance of the Schisto-PCR to be very similar in Senegal and in Kenya, despite differences in the level of *Schistosoma* transmission, geographic *S*. *mansoni* strains, co-infecting helminths, and demographic composition as well as genetic background of the study population. An additional advantage of PCR is that it is more objective and uniform than microscopy. It does not suffer from methodological variations (e.g. number and volume of stool samples, calculation of average egg count, quality of microscopy), or inter-observer variation, and it is less error-prone. Moreover, stool samples can be stored for later analysis by PCR and if needed, in a central laboratory. Hence, the Schisto-PCR may be particularly useful as an epidemiological tool to reliably compare levels of infection between geographical areas and between studies [42]. In addition, PCR can be used as a reference standard to assess the quality of locally used (reference) methods, and to compare the accuracy of diagnostic procedures between different study sites [43].

Multiplex PCR allows the detection of multiple helminth species, and this spectrum can be further expanded by combining different multiplex PCRs such as the Schisto-PCR and ANAS-PCR. In the present study, the ANAS-PCR confirmed microscopy results showing relatively low levels of STH infections. While microscopy and PCR gave similar results for *A*. *lumbricoides*, PCR was more sensitive in the detection of *N*. *americanus* than microscopy. These results are in accordance with previous studies that suggested multiplex PCR to be more sensitive than, or as sensitive as, microscopic techniques for the detection of hookworm and *A*. *lumbricoides* in areas of low STH transmission [44–46]. Additional advantages of the ANAS-PCR are that it can also detect *S*. *stercoralis* and that it can differentiate between the two common hookworm species *N*. *americanus* and *A*. *duodenale*. Very recently, our group further extended the Schisto- and ANAS- multiplex PCRs to include *T*. *trichiura*. In the near future, it will thus be possible to detect not only *Schistosoma* spp. but also the other most important intestinal helminths–*A*. *lumbricoides*, *N*. *americanus*, *A*. *duodenale*, *S*. *stercoralis*, *and T*. *trichiura* [47]–in one single analysis. This is not possible by microscopy.

## Conclusion

In this study, we extensively evaluated the ITS2-based Schisto-PCR on stool samples for the detection of *S*. *mansoni* and showed that it outperforms standard microscopy on Kato-Katz smears. The Schisto-PCR was more sensitive in detecting *S*. *mansoni* than standard microscopy, which makes it particularly useful in low transmission areas, and consequently, in post-control settings. As such, it can be used in the context of schistosomiasis control and elimination, but also for epidemiological research, and for quality control of microscopy. Moreover, it can be complemented with other PCRs such as the ANAS-PCR to detect a wider range of helminths. In this way, DNA-based diagnostic tools may aid in enhancing effectiveness of current integrated NTD control and elimination.

## Supporting Information

S1 STARD Checklist (DOC)

S1 Dataset (XLSX)

S1 SPSS Syntax (DOCX)
