# Supplementary material for: Is PCR the Next Reference Standard for the Diagnosis of Schistosoma in Stool? A Comparison with Microscopy in Senegal and Kenya
Source: PLoS Negl Trop Dis. 2015 Jul 28;9(7):e0003959. doi: 10.1371/journal.pntd.0003959 (PMC4517772; doi:10.1371/journal.pntd.0003959)
Supplement: S1 SPSS Syntax — (DOCX) [file pntd.0003959.s003.docx]

*SENEGAL

*New variables.

COMPUTE logepg_totall=LG10(epg+5).

COMPUTE logepg_totpos=LG10(epg).

EXECUTE.

RECODE epg1 (0=0) (SYSMIS=SYSMIS) (MISSING=SYSMIS) (1 thru Highest=1) INTO Sm_S1.

EXECUTE.

RECODE epg2 (0=0) (SYSMIS=SYSMIS) (MISSING=SYSMIS) (1 thru Highest=1) INTO Sm_S2.

EXECUTE.

COMPUTE Sm1Sh2_S1=Sm_S1+Sh2.

EXECUTE.

IF (epg1>-1)epg1Cat=0.

IF (epg1>0)epg1Cat=1.

IF (epg1>=100)epg1Cat=2.

IF (epg1>=400)epg1Cat=3.

EXECUTE.

IF (S1Ngary_parasites > -1 | S2Ngary_parasites > -1 | S1Yague_parasites > -1 | S2Yague_parasites > -1) tricho=0.

IF (ID>10000 & S1Ngary_eggcount>-1) tricho=0.

IF (S1Ngary_parasites =3 | S2Ngary_parasites =3 | S1Yague_parasites =3 | S2Yague_parasites =3) tricho =3.

IF (S1Ngary_parasites > -1 | S2Ngary_parasites > -1 | S1Yague_parasites > -1 | S2Yague_parasites > -1) ascaris=0.

IF (ID>10000 & S1Ngary_eggcount>-1) ascaris=0.

IF (S1Ngary_parasites =4 | S2Ngary_parasites =4 | S1Yague_parasites =4 | S2Yague_parasites =4) ascaris=4.

IF (S1Ngary_parasites =5 | S2Ngary_parasites =5 | S1Yague_parasites =5 | S2Yague_parasites =5) tricho=3.

IF (S1Ngary_parasites =5 | S2Ngary_parasites =5 | S1Yague_parasites =5 | S2Yague_parasites =5) ascaris=4.

EXECUTE.

*Table 1.

FREQUENCIES VARIABLES= Sm1 Sm_S1 Sm_S2 pcr_sch

/ORDER=ANALYSIS.

USE ALL.

COMPUTE filter_$=(Sm1=1).

VALUE LABELS filter_$ 0 'Not Selected' 1 'Selected'.

FORMATS filter_$ (f1.0).

FILTER BY filter_$.

EXECUTE.

EXAMINE VARIABLES=epg

/STATISTICS DESCRIPTIVES

/MISSING PAIRWISE

/NOTOTAL.

USE ALL.

COMPUTE filter_$=(Sm_S1=1).

VALUE LABELS filter_$ 0 'Not Selected' 1 'Selected'.

FORMATS filter_$ (f1.0).

FILTER BY filter_$.

EXECUTE.

EXAMINE VARIABLES=epg1

/STATISTICS DESCRIPTIVES

/MISSING PAIRWISE

/NOTOTAL.

USE ALL.

COMPUTE filter_$=(pcr_sch=1).

VALUE LABELS filter_$ 0 'Not Selected' 1 'Selected'.

FORMATS filter_$ (f1.0).

FILTER BY filter_$.

EXECUTE.

EXAMINE VARIABLES=ct_sch

/STATISTICS DESCRIPTIVES

/MISSING PAIRWISE

/NOTOTAL.

FILTER OFF.

USE ALL.

EXECUTE.

*Table 2.

CROSSTABS

/TABLES=Sm_S1 BY pcr_sch

/FORMAT=AVALUE TABLES

/CELLS=COUNT ROW COLUMN TOTAL

/STATISTICS=KAPPA

/COUNT ROUND CELL.

CROSSTABS

/TABLES=Sm1 BY pcr_sch

/FORMAT=AVALUE TABLES

/CELLS=COUNT ROW COLUMN TOTAL

/STATISTICS=KAPPA

/COUNT ROUND CELL.

USE ALL.

COMPUTE filter_$=(pcr_sch=1).

VALUE LABELS filter_$ 0 'Not Selected' 1 'Selected'.

FORMATS filter_$ (f1.0).

FILTER BY filter_$.

EXECUTE.

EXAMINE VARIABLES=ct_sch BY Sm_S1

/PLOT BOXPLOT HISTOGRAM NPPLOT

/COMPARE VARIABLES

/PERCENTILES(5,10,25,50,75,90,95) HAVERAGE

/STATISTICS DESCRIPTIVES

/CINTERVAL 95

/MISSING LISTWISE

/NOTOTAL.

NPTESTS

/INDEPENDENT TEST (ct_sch) GROUP (Sm_S1)

/MISSING SCOPE=ANALYSIS USERMISSING=EXCLUDE

/CRITERIA ALPHA=0.05 CILEVEL=95.

EXAMINE VARIABLES=ct_sch BY Sm1

/PLOT BOXPLOT HISTOGRAM NPPLOT

/COMPARE VARIABLES

/PERCENTILES(5,10,25,50,75,90,95) HAVERAGE

/STATISTICS DESCRIPTIVES

/CINTERVAL 95

/MISSING LISTWISE

/NOTOTAL.

NPTESTS

/INDEPENDENT TEST (ct_sch) GROUP (Sm1)

/MISSING SCOPE=ANALYSIS USERMISSING=EXCLUDE

/CRITERIA ALPHA=0.05 CILEVEL=95.

EXAMINE VARIABLES=ct_sch

/COMPARE VARIABLES

/PERCENTILES(5,10,25,50,75,90,95) HAVERAGE

/STATISTICS DESCRIPTIVES

/CINTERVAL 95

/MISSING LISTWISE

/NOTOTAL.

FILTER OFF.

USE ALL.

EXECUTE.

*Fig 1.

NPTESTS

/INDEPENDENT TEST (ct_sch) GROUP (epgCat) KRUSKAL_WALLIS(COMPARE=PAIRWISE) JONCKHEERE_TERPSTRA(ORDER=aSCENDING COMPARE=PAIRWISE)

/MISSING SCOPE=ANALYSIS USERMISSING=EXCLUDE

/CRITERIA ALPHA=0.05 CILEVEL=95.

NPTESTS

/INDEPENDENT TEST (ct_sch) GROUP (epg1Cat) KRUSKAL_WALLIS(COMPARE=PAIRWISE) JONCKHEERE_TERPSTRA(ORDER=aSCENDING COMPARE=PAIRWISE)

/MISSING SCOPE=ANALYSIS USERMISSING=EXCLUDE

/CRITERIA ALPHA=0.05 CILEVEL=95.

CROSSTABS

/TABLES=epgCat epg1Cat BY pcr_sch

/FORMAT=AVALUE TABLES

/CELLS=COUNT

/COUNT ROUND CELL.

*Correlations between egg- and DNA-based infection intensities.

NONPAR CORR

/VARIABLES=epg1 ct_sch

/PRINT=SPEARMAN TWOTAIL NOSIG

/MISSING=PAIRWISE.

NONPAR CORR

/VARIABLES=epg ct_sch

/PRINT=SPEARMAN TWOTAIL NOSIG

/MISSING=PAIRWISE.

GRAPH

/SCATTERPLOT(BIVAR)=epg1 WITH ct_sch

/MISSING=LISTWISE.

GRAPH

/SCATTERPLOT(BIVAR)=epg WITH ct_sch

/MISSING=LISTWISE .

*Table 3.

FREQUENCIES VARIABLES= Sm1Sh2

/ORDER=ANALYSIS.

CROSSTABS

/TABLES=pcr_sch BY Sm1Sh2

/FORMAT=AVALUE TABLES

/STATISTICS=CHISQ

/CELLS=COUNT

/COUNT ROUND CELL.

CROSSTABS

/TABLES=pcr_sch BY SmSh9

/FORMAT=AVALUE TABLES

/STATISTICS=CHISQ

/CELLS=COUNT

/COUNT ROUND CELL.

COMPUTE filter_$=(pcr_sch=1).

VALUE LABELS filter_$ 0 'Not Selected' 1 'Selected'.

FORMATS filter_$ (f1.0).

FILTER BY filter_$.

EXECUTE.

EXAMINE VARIABLES=ct_sch BY Sm1Sh2

/COMPARE VARIABLES

/PERCENTILES(5,10,25,50,75,90,95) HAVERAGE

/STATISTICS DESCRIPTIVES

/CINTERVAL 95

/MISSING LISTWISE

/NOTOTAL.

EXECUTE.

EXAMINE VARIABLES=ct_sch BY SmSh9

/COMPARE VARIABLES

/PERCENTILES(5,10,25,50,75,90,95) HAVERAGE

/STATISTICS DESCRIPTIVES

/CINTERVAL 95

/MISSING LISTWISE

/NOTOTAL.

EXECUTE.

USE ALL.

COMPUTE filter_$=(Sm1=0).

VALUE LABELS filter_$ 0 'Not Selected' 1 'Selected'.

FORMATS filter_$ (f1.0).

FILTER BY filter_$.

EXECUTE.

CROSSTABS

/TABLES=pcr_sch BY Sh2

/FORMAT=AVALUE TABLES

/STATISTICS=CHISQ KAPPA

/CELLS=COUNT

/COUNT ROUND CELL.

USE ALL.

COMPUTE filter_$=(Sm1=0 & pcr_sch=1).

VALUE LABELS filter_$ 0 'Not Selected' 1 'Selected'.

FORMATS filter_$ (f1.0).

FILTER BY filter_$.

EXECUTE.

NPTESTS

/INDEPENDENT TEST (ct_sch) GROUP (Sh2)

/MISSING SCOPE=ANALYSIS USERMISSING=EXCLUDE

/CRITERIA ALPHA=0.05 CILEVEL=95.

COMPUTE filter_$=(Sm1=1).

VALUE LABELS filter_$ 0 'Not Selected' 1 'Selected'.

FORMATS filter_$ (f1.0).

FILTER BY filter_$.

EXECUTE.

CROSSTABS

/TABLES=pcr_sch BY Sh2

/FORMAT=AVALUE TABLES

/STATISTICS=CHISQ KAPPA

/CELLS=COUNT

/COUNT ROUND CELL.

USE ALL.

COMPUTE filter_$=(Sm1=1 & pcr_sch=1).

VALUE LABELS filter_$ 0 'Not Selected' 1 'Selected'.

FORMATS filter_$ (f1.0).

FILTER BY filter_$.

EXECUTE.

NPTESTS

/INDEPENDENT TEST (ct_sch) GROUP (Sh2)

/MISSING SCOPE=ANALYSIS USERMISSING=EXCLUDE

/CRITERIA ALPHA=0.05 CILEVEL=95.

FILTER OFF.

USE ALL.

EXECUTE.

*Table 4.

COMPUTE filter_$=(Sm1=1 & pcr_sch=1).

VALUE LABELS filter_$ 0 'Not Selected' 1 'Selected'.

FORMATS filter_$ (f1.0).

FILTER BY filter_$.

EXECUTE.

SORT CASES BY epgCat.

SPLIT FILE LAYERED BY epgCat.

EXAMINE VARIABLES=ct_sch BY Sh2

/COMPARE VARIABLES

/PERCENTILES(5,10,25,50,75,90,95) HAVERAGE

/STATISTICS DESCRIPTIVES

/CINTERVAL 95

/MISSING LISTWISE

/NOTOTAL.

NPTESTS

/INDEPENDENT TEST (ct_sch) GROUP (Sh2)

/MISSING SCOPE=ANALYSIS USERMISSING=EXCLUDE

/CRITERIA ALPHA=0.05 CILEVEL=95.

SPLIT FILE OFF.

NPTESTS

/INDEPENDENT TEST (ct_sch) GROUP (Sh2)

/MISSING SCOPE=ANALYSIS USERMISSING=EXCLUDE

/CRITERIA ALPHA=0.05 CILEVEL=95.

*____________________________________________________________________________________________________________________

*KENYA

*New variables.

COMPUTE logepg_totall=LG10(epg_m+1).

COMPUTE logepg_totpos=LG10(epg_m).

EXECUTE.

COMPUTE epg_m2=MEAN(k2a, k2b).

VARIABLE LABELS epg_m2 'Stool 2 mean Sm eggs per gram'.

EXECUTE.

COMPUTE epg_m3=MEAN(k3a, k3b).

VARIABLE LABELS epg_m3 'Stool 3 mean Sm eggs per gram'.

EXECUTE.

COMPUTE epg_m12=MEAN(k1a, k1b, k2a, k2b).

VARIABLE LABELS epg_m12 'Stool 1+2 mean Sm eggs per gram'.

EXECUTE.

RECODE epg_m1 (0=0) (SYSMIS=SYSMIS) (MISSING=SYSMIS) (1 thru Highest=1) INTO Sm_S1.

EXECUTE.

RECODE epg_m2 (0=0) (SYSMIS=SYSMIS) (MISSING=SYSMIS) (1 thru Highest=1) INTO Sm_S2.

EXECUTE.

RECODE epg_m3 (0=0) (SYSMIS=SYSMIS) (MISSING=SYSMIS) (1 thru Highest=1) INTO Sm_S3.

EXECUTE.

RECODE epg_m12 (0=0) (SYSMIS=SYSMIS) (MISSING=SYSMIS) (1 thru Highest=1) INTO Sm_S12.

EXECUTE.

IF (epg_m1>-1)epg1Cat=0.

IF (epg_m1>0)epg1Cat=1.

IF (epg_m1>=100)epg1Cat=2.

IF (epg_m1>=400)epg1Cat=3.

EXECUTE.

IF (epg_m>-1)epgCat=0.

IF (epg_m>0)epgCat=1.

IF (epg_m>=100)epgCat=2.

IF (epg_m>=400)epgCat=3.

EXECUTE.

*Table 1.

FREQUENCIES VARIABLES= epg_pos Sm_S12 Sm_S1 Sm_S2 Sm_S3 pcr_sch

/ORDER=ANALYSIS.

USE ALL.

COMPUTE filter_$=(epg_pos=1).

VALUE LABELS filter_$ 0 'Not Selected' 1 'Selected'.

FORMATS filter_$ (f1.0).

FILTER BY filter_$.

EXECUTE.

EXAMINE VARIABLES=epg_m

/STATISTICS DESCRIPTIVES

/MISSING PAIRWISE

/NOTOTAL.

USE ALL.

COMPUTE filter_$=(Sm_S1=1).

VALUE LABELS filter_$ 0 'Not Selected' 1 'Selected'.

FORMATS filter_$ (f1.0).

FILTER BY filter_$.

EXECUTE.

EXAMINE VARIABLES=epg_m1

/STATISTICS DESCRIPTIVES

/MISSING PAIRWISE

/NOTOTAL.

USE ALL.

COMPUTE filter_$=(Sm_S12=1).

VALUE LABELS filter_$ 0 'Not Selected' 1 'Selected'.

FORMATS filter_$ (f1.0).

FILTER BY filter_$.

EXECUTE.

EXAMINE VARIABLES=epg_m12

/STATISTICS DESCRIPTIVES

/MISSING PAIRWISE

/NOTOTAL.

USE ALL.

COMPUTE filter_$=(pcr_sch=1).

VALUE LABELS filter_$ 0 'Not Selected' 1 'Selected'.

FORMATS filter_$ (f1.0).

FILTER BY filter_$.

EXECUTE.

EXAMINE VARIABLES=ct_sch

/STATISTICS DESCRIPTIVES

/MISSING PAIRWISE

/NOTOTAL.

FILTER OFF.

USE ALL.

EXECUTE.

*Table 2.

CROSSTABS

/TABLES=Sm_S1 BY pcr_sch

/FORMAT=AVALUE TABLES

/STATISTICS=KAPPA

/CELLS=COUNT ROW COLUMN TOTAL

/COUNT ROUND CELL.

CROSSTABS

/TABLES=Sm_S12 BY pcr_sch

/FORMAT=AVALUE TABLES

/STATISTICS=KAPPA

/CELLS=COUNT ROW COLUMN TOTAL

/COUNT ROUND CELL.

CROSSTABS

/TABLES=epg_pos BY pcr_sch

/FORMAT=AVALUE TABLES

/STATISTICS=KAPPA

/CELLS=COUNT ROW COLUMN TOTAL

/COUNT ROUND CELL.

USE ALL.

COMPUTE filter_$=(pcr_sch=1).

VALUE LABELS filter_$ 0 'Not Selected' 1 'Selected'.

FORMATS filter_$ (f1.0).

FILTER BY filter_$.

EXECUTE.

EXAMINE VARIABLES=ct_sch BY Sm_S1

/PLOT BOXPLOT HISTOGRAM NPPLOT

/COMPARE VARIABLES

/PERCENTILES(5,10,25,50,75,90,95) HAVERAGE

/STATISTICS DESCRIPTIVES

/CINTERVAL 95

/MISSING LISTWISE

/NOTOTAL.

NPTESTS

/INDEPENDENT TEST (ct_sch) GROUP (Sm_S1)

/MISSING SCOPE=ANALYSIS USERMISSING=EXCLUDE

/CRITERIA ALPHA=0.05 CILEVEL=95.

EXAMINE VARIABLES=ct_sch BY Sm_S12

/PLOT BOXPLOT HISTOGRAM NPPLOT

/COMPARE VARIABLES

/PERCENTILES(5,10,25,50,75,90,95) HAVERAGE

/STATISTICS DESCRIPTIVES

/CINTERVAL 95

/MISSING LISTWISE

/NOTOTAL.

NPTESTS

/INDEPENDENT TEST (ct_sch) GROUP (Sm_S12)

/MISSING SCOPE=ANALYSIS USERMISSING=EXCLUDE

/CRITERIA ALPHA=0.05 CILEVEL=95.

EXAMINE VARIABLES=ct_sch BY epg_pos

/PLOT BOXPLOT HISTOGRAM NPPLOT

/COMPARE VARIABLES

/PERCENTILES(5,10,25,50,75,90,95) HAVERAGE

/STATISTICS DESCRIPTIVES

/CINTERVAL 95

/MISSING LISTWISE

/NOTOTAL.

NPTESTS

/INDEPENDENT TEST (ct_sch) GROUP (epg_pos)

/MISSING SCOPE=ANALYSIS USERMISSING=EXCLUDE

/CRITERIA ALPHA=0.05 CILEVEL=95.

EXAMINE VARIABLES=ct_sch

/COMPARE VARIABLES

/PERCENTILES(5,10,25,50,75,90,95) HAVERAGE

/STATISTICS DESCRIPTIVES

/CINTERVAL 95

/MISSING LISTWISE

/NOTOTAL.

FILTER OFF.

USE ALL.

EXECUTE.

*Fig 1.

*Nonparametric Tests: Independent Samples.

NPTESTS

/INDEPENDENT TEST (ct_sch) GROUP (epgCat) KRUSKAL_WALLIS(COMPARE=PAIRWISE) JONCKHEERE_TERPSTRA(ORDER=DESCENDING COMPARE=PAIRWISE)

/MISSING SCOPE=ANALYSIS USERMISSING=EXCLUDE

/CRITERIA ALPHA=0.05 CILEVEL=95.

NPTESTS

/INDEPENDENT TEST (ct_sch) GROUP (epg1Cat) KRUSKAL_WALLIS(COMPARE=PAIRWISE) JONCKHEERE_TERPSTRA(ORDER=DESCENDING COMPARE=PAIRWISE)

/MISSING SCOPE=ANALYSIS USERMISSING=EXCLUDE

/CRITERIA ALPHA=0.05 CILEVEL=95.

CROSSTABS

/TABLES=epgCat epg1Cat BY pcr_sch

/FORMAT=AVALUE TABLES

/CELLS=COUNT

/COUNT ROUND CELL.

*Correlations between egg- and DNA-based infection intensities.

NONPAR CORR

/VARIABLES=epg_m1 ct_sch

/PRINT=SPEARMAN TWOTAIL NOSIG

/MISSING=PAIRWISE.

NONPAR CORR

/VARIABLES=epg_m12 ct_sch

/PRINT=SPEARMAN TWOTAIL NOSIG

/MISSING=PAIRWISE.

NONPAR CORR

/VARIABLES=epg_m ct_sch

/PRINT=SPEARMAN TWOTAIL NOSIG

/MISSING=PAIRWISE.

GRAPH

/SCATTERPLOT(BIVAR)=epg_m1 WITH ct_sch

/MISSING=LISTWISE.

GRAPH

/SCATTERPLOT(BIVAR)=epg_m WITH ct_sch

/MISSING=LISTWISE.

*Fig 2.

DATASET DECLARE school.

AGGREGATE

/OUTFILE='school'

/BREAK=school

/prev_zone_first=FIRST(prevalence_zone)

/Sm_S1_pgt=PGT(Sm_S1 0)

/epg_pos_pgt=PGT(epg_pos 0)

/pcr_sch_pgt=PGT(pcr_sch 0)

/ct_sch_median=MEDIAN(ct_sch)

/N_BREAK=N.

EXECUTE.

COMPUTE filter_$=(N_BREAK>14).

VALUE LABELS filter_$ 0 'Not Selected' 1 'Selected'.

FORMATS filter_$ (f1.0).

FILTER BY filter_$.

EXECUTE.

GRAPH

/SCATTERPLOT(OVERLAY)=Sm_S1_pgt epg_pos_pgt WITH pcr_sch_pgt pcr_sch_pgt (PAIR)

/MISSING=LISTWISE.

CORRELATIONS

/VARIABLES=Sm_S1_pgt epg_pos_pgt pcr_sch_pgt

/PRINT=TWOTAIL NOSIG

/MISSING=PAIRWISE.

NONPAR CORR

/VARIABLES=Sm_S1_pgt epg_pos_pgt pcr_sch_pgt

/PRINT=SPEARMAN TWOTAIL NOSIG

/MISSING=PAIRWISE.
